# Advances in the Diagnosis and Treatment of Non-Alcoholic Fatty Liver Disease

**DOI:** 10.3390/ijms24032844

**Published:** 2023-02-02

**Authors:** Xunzhe Yin, Xiangyu Guo, Zuojia Liu, Jin Wang

**Affiliations:** 1 State Key Laboratory of Electroanalytical Chemistry, Changchun Institute of Applied Chemistry, Chinese Academy of Sciences, Changchun 130022, China; 2 Department of Chemistry and Physics, Stony Brook University, Stony Brook, New York, NY 11794-3400, USA

**Keywords:** NAFLD, pathogenesis, diagnosis, treatment

## Abstract

Non-alcoholic fatty liver disease (NAFLD) is the most prevalent chronic liver disease that affects approximately one-quarter of the global adult population, posing a significant threat to human health with wide-ranging social and economic implications. The main characteristic of NAFLD is considered that the excessive fat is accumulated and deposited in hepatocytes without excess alcohol intake or some other pathological causes. NAFLD is a progressive disease, ranging from steatosis to non-alcoholic steatohepatitis (NASH), cirrhosis, hepatocellular carcinoma, liver transplantation, and death. Therefore, NAFLD will probably emerge as the leading cause of end-stage liver disease in the coming decades. Unlike other highly prevalent diseases, NAFLD has received little attention from the global public health community. Liver biopsy is currently considered the gold standard for the diagnosis and staging of NAFLD because of the absence of noninvasive and specific biomarkers. Due to the complex pathophysiological mechanisms of NAFLD and the heterogeneity of the disease phenotype, no specific pharmacological therapies have been approved for NAFLD at present, although several drugs are in advanced stages of development. This review summarizes the current evidence on the pathogenesis, diagnosis and treatment of NAFLD.

## 1. Introduction

Non-alcoholic fatty liver disease (NAFLD) represents a serious liver pathology, which requires high health care costs, causes economic losses and reduces health-related quality of life [1,2,3]. The main feature of NAFLD is hepatic steatosis, which is currently defined in the NAFLD management’s guidelines as steatosis with ≥5% liver fat detected by imaging techniques or histology in patients with no other chronic liver disease consuming little to no alcohol [4,5]. NAFLD is a general term utilized for a wide range of clinicopathological findings. Histologically, NAFLD is a disease continuum including steatosis accompanied by mild inflammation or not (non-alcoholic fatty liver, NAFL) and NASH, which in addition features hepatocellular damage (hepatocyte ballooning) [6]. With the rising prevalence rates of metabolic disorders, including diabetes, obesity, dyslipidemia and metabolic syndrome, NAFLD incidence is growing year by year, which further increases the odds of cardiovascular disease and death. Meanwhile, NAFLD also increases the odds of liver cancer, and is considered the primary cause of liver cancer in the Western world [7]. Therefore, the early detection, diagnosis and treatment of NAFLD has become urgent. The present review describes current findings about the pathogenesis of NAFLD and highlights progress in its diagnosis and treatment, which may help improve the outcomes of NAFLD cases.

## 2. Definition

**Figure 1:**
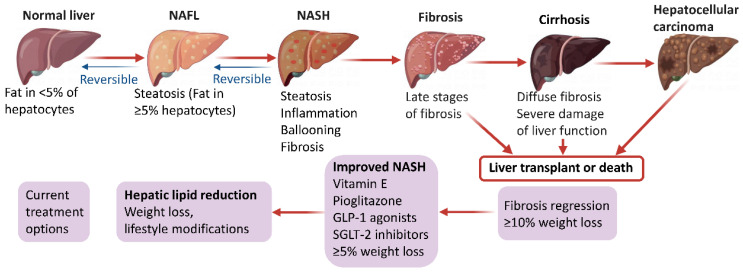
Pathophysiology and management options in NAFLD.

NAFLD begins with ectopic fat buildup in the liver and further manifests as an impaired stress response resulting from an excessive supply of nutrients, mainly comprising glucose, fructose and fatty acids. Under normal conditions, fatty acid supply to the liver relies on the degradation of triglycerides (TGs) in fat tissue. Subsequently, the liver eliminates fatty acids to generate inert TGs that enter the circulation in the form of very low-density lipoprotein or transiently remain in liver cells as lipid droplets. During this process, fatty acids can provide energy through β-oxidation in the mitochondria or peroxisomes [8]. However, de novo lipogenesis (DNL) converts excess carbohydrates (e.g., fructose, glucose) into fatty acids and excess energy is stored as lipids in a disorderly manner in the liver, which is approximately the main fatty acid supply to the liver in NAFLD cases [6,9,10]. Once fatty acids are excessively produced via DNL or adipose lipolysis, or in the case of impaired or overwhelmed fatty acid oxidation, they can generate lipotoxic substances, including lysophosphatidylcholine, lysophosphatidic acid, ceramides, and diacylglycerols [11,12]. Prolonged lipotoxicity induces endoplasmic reticulum stress, inflammation, mitochondrial dysfunction, hepatocyte damage, and the production of profibrogenic stimuli to hepatic stellate cells. [13,14]. Jointly, these events finally induce the histological features of NAFLD, including progressive liver NASH, fibrosis, cirrhosis, and hepatocellular carcinoma (Figure 1). Interestingly, based on the diverse metabolic events involved in NAFLD development and progression, several expert panels recently suggested to change the disease name from NAFLD to metabolic (dysfunction)-associated fatty liver disease (MAFLD) [15,16,17,18]. This novel definition of NAFLD may increase awareness and research of the disease, and effectively accelerate the translational path to new treatments.

## 3. The Mechanism of NAFLD

### 3.1. Pathogenesis

**Figure 2:**
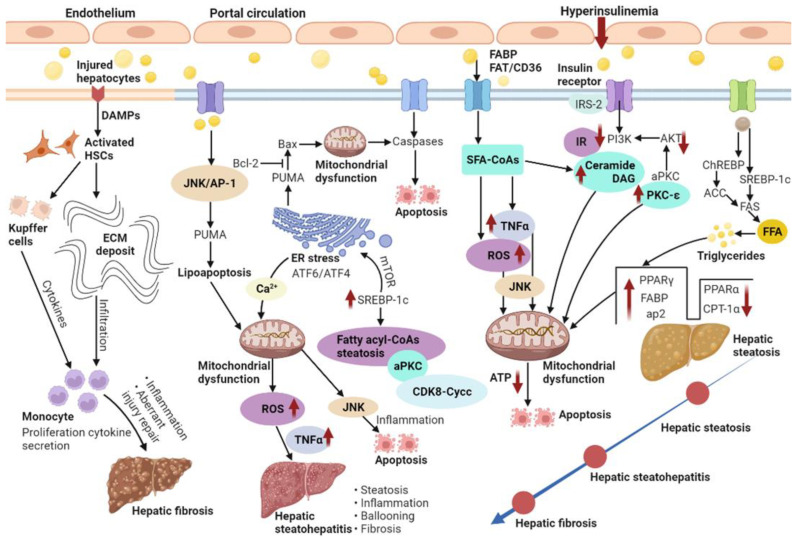
NAFLD is characterized by the excess accumulation of TG in the hepatocytes due to the increased inflow of both FFAs and DNL. Additionally, NASH and fibrosis is also present.

NAFLD features a buildup of fats such as TGs in hepatocytes. The occurrence of hepatic steatosis in most individuals depends on ingested dietary fats [19]. A reasonable enlargement of visceral fat tissue co-occurs with free fatty acid (FFA) secretion into the portal circulation, from which FFAs translocate into the liver to exert lipotoxicity [20]. Therefore, NAFLD features excessive TG buildup in hepatocytes both because of enhanced inflow of FFAs and DNL [21]. When the supply of fatty acids surpasses the ability of β-oxidation, accumulated acyl-CoA is utilized for TG biosynthesis, inducing liver steatosis. Indeed, fatty acid or fatty acyl-CoA buildup is more detrimental to the liver compared with TG accumulation [22]. Usually, about 60% of the liver TG content in NAFLD cases isderived from circulating non-esterified fatty acids, and the remaining accounts for 40% from DNL or the diet [23]. Thus, DNL is substantially important in the pathogenetic mechanism of NAFLD. In DNL, glucose is transformed into acetyl CoA via glycolysis and pyruvate oxidation. Acetyl-CoA is next transformed into malonyl-CoA by acetyl-CoA carboxylase (ACC). Fatty acid synthase (FAS) catalyzes palmitic acid generation from malonyl-CoA and acetyl-CoA. Glucose and insulin induce lipogenesis by activating carbohydrate-response element-binding protein (ChREBP) and sterol regulatory element-binding protein-1c (SREBP-1c), respectively (Figure 2). Furthermore, ChREBP enhances its interaction with carbohydrate responsive element in the promoter regions of glycolytic and lipogenic genes such as liver-pyruvate kinase, ACC, and FAS [9,24].

Fatty acid-binding proteins (FABPs) belong to the superfamily of lipid-binding proteins [25]. In addition to FABPs, fatty acid translocation mostly utilizes three additional fatty acid transporters, i.e., fatty acid transport proteins (FATP), fatty acid translocase (FAT/CD36) and caveolin-1. Subsequently, fat buildup as lipid droplets in hepatocytes causes hepatic steatosis [26]. Lipid droplets in cells comprise the molecular machinery for synthesizing, storing, utilizing, and degrading diverse lipid derivatives from the enzymatic activity of ACC [27]. Additionally, lipid droplets’ proteins also affect the pathophysiology of fatty liver disease. Therefore, high fat diet-associated peroxisome proliferator-activated receptor-gamma (PPAR-γ) enhances and develops fatty liver [28]. Liver lipase activity shows a positive correlation with intra-abdominal fat levels; thus, hyperinsulinemia correlates with insulin receptor substrate-2 (IRS-2) downregulation in the liver [29]. First, insulin-dependent induction of tyrosine phosphorylation of IRS-2 is reduced. Subsequently, IRS-2-associated phosphatidylinositol 3-kinase (PI3K) activity is decreased and Akt phosphorylation is seriously repressed (Figure 2). Despite IRS-2 downregulation, insulin continuously increases SREBP-1c amounts. Eventually, glucose overproduction combined with induced fatty acid biosynthesis further increases insulin secretion and resistance in a vicious cycle [30].

NASH features steatosis with diffused lobular inflammation, hepatocellular damage (hepatocyte ballooning as a hallmark), and fibrosis to diverse extents. Liver inflammation drives disease progression, and up to one-third of NASH cases may progress to advanced fibrosis or cirrhosis, which increases liver disease-associated death because of cirrhosis complications, e.g., HCC. At present, the prognostic value of inflammation in NASH considering liver-associated morbidity or mortality remains controversial. A systematic review of trials with paired biopsy specimens indicated that histological inflammation in the first biopsy sample independently predicts the progression to advanced fibrosis in NASH [31]. However, retrospective longitudinal trials reported liver fibrosis, not inflammation, as the major prognostic factor of liver-disease associated mortality [32]. This does not necessarily suggest inflammation and hepatocyte ballooning have no prognostic value, as the natural course of NAFLD may span decades; therefore, detecting the association of NASH with long-term prognosis may require larger and optimally powered long-term trials with strict histological definitions. Inflammation induces the secretion of diverse inflammatory factors, including cytokines, chemokines and eicosanoids, which orchestrate cell defense mechanisms and tissue regeneration. However, inflammation persistence may induce chronic inflammatory alterations, which exacerbate tissue damage and may trigger an abnormal wound-healing response contributing, in NAFLD cases, to the etiology of NASH and liver fibrosis.

Correlating with liver disease progression, organ fibrosis is a characteristic of disease progression in chronic inflammatory disorders, which represents an important factor determining liver disease outcome and HCC occurrence. In the same manner, in the liver, fibrosis greatly affects the patient’s quality of life and prognosis [33]. Liver fibrosis features a continuous extracellular matrix (ECM) buildup that alters the normal liver features. Pathogenetically, liver metabolic disorders damage hepatocytes and alter the infiltration of immune cells that induce the trans-differentiation of hepatic stellate cells (HSCs) into collagen-producing myofibroblasts (Figure 2). In NASH, an impaired balance of pro-fibrogenic and anti-fibrogenic mechanisms results in continuous induction of proliferating, contractile and migrating myofibroblasts, which excessively produce the ECM. The liver’s fate to progress to an anti-fibrotic scar-dissolving phase or an unsuppressed fibrosis-promoting phase is hereby mostly controlled by non-parenchymal cells (NPCs), e.g., Kupffer cells and other immune cells. Therefore, hepatocyte apoptosis and damage-associated pattern (DAMP) release by liver cells both induce HSCs directly and enhance the infiltration and activation of lymphocytes and macrophages, promoting HSC trans-differentiation and myofibroblast activation by generating pro-inflammatory and pro-fibrogenic cytokines [34,35]. At the molecular level, interconnected cytokine-induced signaling pathways are responsible for pro-fibrogenic cell interactions. Indeed, transforming growth factor beta (TGF-β), platelet derived growth factor (PDGF), the inflammasome (NLRP3)-caspase1 pathway, and Wnt/β-catenin signaling are considered the major pathways controlling HSC activation and fibrosis progression.

### 3.2. Insulin Resistance

Insulin resistance (IR) represents the main mechanism underlying the development and progression of NAFLD; IR progression results in continuous lipolysis from insulin-resistant intraabdominal visceral fat accumulation. Consequently, the therapeutic effects of insulin sensitizers on NAFLD/NASH are very important. Indeed, IR is caused by progressive mitochondrial dysfunction, likely constituting the primary event triggering obesity-related NAFLD [36]. IR in NAFLD features decreased whole-body, liver, and fat tissue insulin sensitivity, which might induce liver injury and liver disease progression. According to the “two-hit” hypothesis, the second hit encompasses metabolic oxidative stress-, autophagy- and inflammation-induced NASH progression [37]. Obesity induces chronic inflammation and cytokine release from adipocytes or macrophages infiltrating the fat tissue. As depicted in Figure 2, IR with deficient insulin receptor substrate-2 (IRS-2)-related PI3K activity increases the cell amounts of fatty acid-derived metabolites, including diacylglycerol (DAG), fatty acyl CoA or ceramides [38,39]. A progressive elevation of the mean TG/DAG ratio was detected in normal livers progressing to NAFLD and eventually developing NASH. Since NAFLD has demonstrated associations with obesity and peripheral IR, IR enhances lipolysis and induces FFA delivery to the liver. The last step of liver TG biosynthesis is catalyzed by acyl-coenzyme A:DAG acyltransferase (DGAT) [40]. Thus, overexpression of liver DGAT induces hepatic steatosis. Conversely, DGAT inhibition prevents fat-associated hepatic IR by decreasing hepatic DAG amounts and protein kinase C (PKC) activation via reduced SREBP1c-related lipogenesis and elevated liver fatty acid oxidation [41]. PKC family members control IR occurrence in the liver. An increase in hepatic DAG content has been reported that may activate PKC-ε and repress insulin signaling. In addition, hepatic IR involves ceramide-related atypical protein kinase C (aPKC) activation that specifically alters Akt-associated forkhead box O1 protein (FOXO1) phosphorylation [42]. Liver aPKC activation involves IRS-2/PI3K and is sustained under high-fat diet and obesity. Increased liver aPKC activation in the hyperinsulinemic state may upregulate SREBP-1c [43]. Meanwhile, mammalian target of rapamycin (mTOR) activation also increases SREBP-1c expression, enhancing intrahepatic TG accumulation. In this process, insulin-associated SREBP-1c upregulation and PEPCK downregulation are both inhibited by PI3K and Akt suppressors. Recently, remarkably decreased protein amounts of cyclin-dependent kinase 8 (CDK8) and Cyclin C (CycC) were detected in obesity in comparison with normal livers. In addition, SREBP-1c and CDK8-CycC complex proteins are inversely correlated in human NAFLD. Downregulated CDK8–CycC complex formation by SREBP-1c is a critical factor in liver DNL in NAFLD and hyperinsulinemia [44].

### 3.3. Mitochondrial Dysfunction

Mitochondrial respiratory chain complexes show reduced activities in liver samples from NASH cases, which depicts a negative correlation with serum TNF-α, IR, as well as an elevated body mass index (BMI) [45]. The amounts of hepatic TGs are readily changed due to TGs/FFA partitioning and TGs-FFA metabolism by mitochondrial β-oxidation. The key regulator of fatty acid transport from the cytosol into the mitochondria and the hepatic mitochondrial β-oxidation flux is malonyl-CoA-sensitive carnitine palmitoyl transferase-1 (CPT-1), which oxidizes fatty acids. ACC, an important regulator of DNL, generates malonyl-CoA. In Figure 2, impairment of mitochondrial β-oxidation due to increased PPAR-γ, FABP, activator protein-2 (ap2) and suppressed PPARα and CPT-1α highly contributes to the pathogenetic mechanism of hepatic steatosis [46]. However, PPARα and PPAR-γ have opposite effects on hepatic steatosis, suppressing and favoring lipogenesis, respectively. Hepatic IR contributes to alterations in mitochondrial oxidative capacity, while reduced liver ATP synthesis causes IR [47]. Primary defects in mitochondrial β-oxidation ability are believed to enhance DAG buildup, PKC-ε activation, and hepatic IR, which can also result in excessive reactive oxygen species (ROS) accumulation [48]. Subsequently, ROS-related release of TNFα and FAS induces mitochondrial membrane permeabilization and apoptosis. The generation of ceramides can be mediated by TNFα, which promotes hepatic IR and attenuates Akt signaling. This is critical for the activation of major pathways regulating steatosis, fibrosis and lipotoxicity.

### 3.4. Hepatocyte Lipoapoptosis

Liver cell lipoapoptosis represents an essential property of NASH. Despite similar cell steatosis, treatment with saturated FFAs causes more pronounced apoptosis compared with exposure to unsaturated counterparts [49]. Saturated FFAs activate protein phosphatase 2A through FOXO3a activation. The direct interaction of FOXO3a with the promoter of the intracellular death mediator Bim increases its expression and lipoapoptosis [50]. Additionally, saturated FFAs enhance c-Jun N-terminal kinase (JNK)-associated hepatocyte lipoapoptosis via the activation of proapoptotic Bcl-2 proteins, Bcl-2-interacting mediator of cell death (Bim) and Bax. Of the latter proteins, Bax triggers the intrinsic apoptotic pathway.

An important pro-apoptotic protein, p53 upregulated modulator of apoptosis (PUMA), is involved in FFA-associated lipoapoptosis in hepatocytes via the JNK1/activator protein-1 (AP-1) complex. PUMA enhances Bax translocation to the mitochondria, triggering pro-apoptotic events. JNK1/c-Jun-related PUMA transcriptional upregulation with further Bax activation represents a step in the induction of saturated FFA-mediated apoptosis (Figure 2). Therefore, mitochondria constitute the main regulators of fatty acid removal and induce changes that help hepatocytes counteract excessive fat accumulation. Mitochondrial function impairment is involved in diverse pathogenetic mechanisms of NASH. In this case, ROS-mediated mitochondrial permeability transition might enhance mtDNA release that compromises oxidative phosphorylation and initiates a vicious cycle leading to mitochondrial collapse. Defective mitochondrial oxidative phosphorylation, e.g., via decreased respiratory chain complexes, is prominently found in NASH. In addition, mitochondrial dysfunction influences lipid deposition in liver cells and induces lipid peroxidation, ROS accumulation and cytokine release, promoting inflammation and cell death [51].

### 3.5. Oxidative Stress and Endoplasmic Reticulum Stress

Oxidative stress degrades lipids, proteins and DNA by inducing inflammation, which induces steatosis progression to NASH. It is commonly found in chronic liver disorders and highly contributes to NASH progression [52]. In addition, oxidative stress biomarkers correlate with neutrophil numbers and liver injury extent in human NASH [53]. ROS also induce lysosomal membrane disruption, causing lysosomal membrane permeabilization and protease release into the cytosol, which induces apoptosis as well as necrosis [54]. In the liver, Kupffer cells are the key cells involved in ROS production, which often relies on NADPH oxidase [55]. DAMPs, including ATP, activate Kupffer cells and induce ROS synthesis [56]. Some lipid peroxidation products can activate hepatic stellate cells. In turn, hepatic stellate cells can produce ROS, possessing phagocytotic activity and expressing NADPH oxidase. Further, cytochrome P450 2E1 (CYP2E1) is a microsomal enzyme involved in fatty acid oxidation and ROS production. Besides, its expression is elevated in NASH models, promoting oxidative damage in hepatocytes [57]. It was reported that increased oxidative stress causes apoptosis, inflammation and fibrogenesis, due to increased auto-oxidation of excess cholesterol-generating oxysterols in clinical biopsy-confirmed NAFLD [58]. Endoplasmic reticulum (ER) stress signaling highly contributes to lipogenesis attenuation and cell protection by enhancing transcription factor 6 (ATF6) expression and transcription factor 4 (ATF4) activation, respectively (Figure 2). During ER stress, Ca^2+^ from the ER is taken up by mitochondria and mitochondrial ROS accumulation increases. Ca^2+^ uptake triggers the opening of transition pores and cytochrome *c* release, which results in defective mitochondrial oxidative function and increased ROS production [59]. JNK signaling is activated by both oxidative stress and ER stress, as a major mediator of IR and fatty acid-related hepatotoxicity.

## 4. Diagnosis of NAFLD

With Westernized eating habits and urbanization, NAFLD prevalence is higher than previously predicted, with a quickly rising trend globally. Therefore, developing reliable and practical tools for diagnosing NAFLD during disease screening is very important in the early detection and effective therapy of NAFLD. Although simple steatosis cases have relatively low odds of developing NAFLD, early diagnosis may help apply appropriate prevention tools to prevent advanced liver diseases. Liver biopsy is not applicable to diagnose simple steatosis due to its asymptomatic nature and hardly understood clinical grounds. Therefore, simple and reproducible biomarkers and scoring systems are needed for hepatic steatosis diagnosis. Biomarker panels currently utilized include two categories: serum and imaging biomarkers. Usually, there is a limitation that not all biomarkers are strictly specific to the patients’ livers and, thus, the presence or absence of other conditions must be considered. However, these limitations may be partially overcome by imaging tools for assessing hepatic steatosis, including magnetic resonance- and ultrasound-based diagnostic tools, which can directly reveal the liver’s intrinsic properties of texture or stiffness. Here we will discuss the current diagnostic methods for NAFLD.

### 4.1. Noninvasive Diagnosis of Steatosis

#### 4.1.1. Fatty Liver Index (FLI)

The FLI represents a noninvasive and well-predictive algorithm for the estimation of hepatic steatosis, which is broadly utilized clinically because of simplicity. The established method relies on BMI, waist circumference, serum TG and gamma-glutamyl transferase (GGT) measurements [60]. In the latter algorithm, an FLI score <30 excludes fatty liver; scores between 30 and 60 indicate uncertain condition, whereas a score ≥60 suggests a definite prediction of hepatic steatosis development [60]. Its main disadvantages are the lack of prediction accuracy and the inability to reflect risk classification. However, overall, raising awareness of FLI in individuals with suspected hepatic steatosis may likely provide novel tools for clinical management and counseling.

#### 4.1.2. NAFLD Liver Fat Score (NLFS)

The NLFS was developed based on proton magnetic resonance spectroscopy (H-MRS) to predict NAFLD and measure liver fat content as a reference standard for hepatic steatosis [61]. When compared to other scores to detect hepatic steatosis, this score mainly includes AST, AST/ALT ratio, and fasting serum insulin level and is usually assessed as liver fat >5.56% (sensitivity, 86% and specificity, 71%). The NLFS allows the identification of NAFLD using easily available clinical and laboratory data, which are thus inexpensive tools that help in predicting patients who are at increased risk of NAFLD.

#### 4.1.3. Lipid Accumulation Product (LAP)

The LAP, initially developed for the U.S. National Health and Nutrition Examination Survey, is considered a biomarker of central obesity [62]. The LAP was designed for identifying fatty liver disease and determining cardiovascular risk, separating patients with no, intermediate and severe fatty liver using ultrasound images. Therefore, this index is closely associated with the presence and severity of NAFLD, which has been recognized as the liver manifestation of metabolic syndrome [63]. In recent years, several studies have also supported the description of lipid accumulation through the utilization of the LAP, for the screening of metabolic syndrome in healthy and patient populations [64,65]. However, the LAP only uses fasting TG levels and waist circumference with gender-specific cut-offs compared to the FLI [66]. Although this score is straightforward and simple, it has not been widely adopted because waist circumference measurements are not standardized or a robust variable. Recent studies have reported it is hard to comparatively assess diagnostic values directly in independent validations of FLI, NLFS, and LAP, as diverse panels use various reference standards [67,68,69]. Collectively, the main limitation of the wide application of assays for detecting hepatic steatosis is related to the lack of pharmacotherapeutic and patientcare pathways.

#### 4.1.4. Novel NAFLD Biomarkers

Because liver biopsy has important risks and cannot be applied broadly, identifying novel noninvasive biomarkers for simple steatosis, NASH and fibrosis is very important. Thrombospondin 2 (TSP-2), belonging to the thrombospondin family, represents a matricellular glycoprotein interacting with the extracellular matrix structural proteins, cellular receptors, growth factors, and cytokines [70]. Recent studies have reported that serum TSP-2 amounts are moderately correlated with ballooning and fibrosis degree, and represent a potential prognostic molecular marker for clinical diagnosing NASH and advanced fibrosis in NAFLD [71,72,73]. Omics technologies can identify new markers with usefulness in NAFLD diagnosis through the detection of thousands of metabolites [74]. Metabolomics has been used to determine the metabolic profiles specific to steatosis and NASH. Particularly, arachidonic acid oxidation products are considered biomarkers of NASH [74]. Nevertheless, omics studies require validation in cohorts with larger sample sizes and higher heterogeneity before clinical application. Currently, non-coding RNA can serve as an emerging non-invasive biomarker for NAFLD detection. An important property making them excellent molecular markers is their high stability. In the general population, circulating RNAs can be used as a NAFLD biomarker. miR-122 plays an essential role in NAFL/NASH’s differential diagnosis and fibrosis stage definition [75,76]. We summarize the currently recommended biomarkers as listed in Table 1.

### 4.2. Imaging for Hepatic Steatosis

#### 4.2.1. Ultrasonography

Routine ultrasonography is broadly utilized for the diagnosis of steatosis, which presents as a typical hyperechoic liver. Ultrasound is commonly applied clinically because of its simplicity, cost-effectiveness and ease of operation, and steatosis may be individually categorized as mild, moderate or severe, by ultrasound analysis. A recent meta-analysis showed that compared to histology (gold standard), ultrasonography is accurate and reliable in detecting fatty liver, with a pooled sensitivity and specificity of 84.8% and 93.6%, respectively, for identifying ≥20–30% steatosis, and an overall area under the receiver operating characteristic curve (AUROC) of 0.93 [77]. Nevertheless, conventional ultrasonography is limited to detecting ≥20% liver fat whereas steatosis cases starting from 5% liver fat are omitted. In addition, its accuracy is also reduced in detecting liver fat in obese and severe fibrotic patients. To cope with these limitations, some ultrasonography-based scoring systems have been developed, with a higher sensitivity in identifying steatosis <20% liver fat. An ultrasonography-based scoring system developed in Japan showed a high accuracy, with elevated pooled sensitivity (91.2–92.6%) and specificity (100%) in non-alcoholic individuals [78]. After the semi-quantitative and quantitative scoring systems were developed, both demonstrated better diagnostic performances than conventional ultrasonography in detecting steatosis in overweight and obese individuals. The most important difference between them was that the quantitative approach (sensitivity of 95% and specificity of 100%) uses computer-based ultrasonography hepatic/renal ratio and hepatic attenuation rate [79,80]. Moreover, ultrasonography can also be combined with the noninvasive algorithm FLI to accurately determine mild to moderate hepatic steatosis, with significant correlations with histological indexes in populations [81,82,83]. Recent studies have also reported several novel diagnostic tools for the analysis of ultrasonography images based on deep learning algorithms, which have been promising in fatty liver assessments [84,85,86]. As a result, ultrasonography is considered the preferred diagnostic tool in people with suspected NAFLD in clinical settings, which may help better identify the determinants of fatty liver disease and intervene directly for reducing the clinical complications of NAFLD.

#### 4.2.2. Computer Tomography (CT)

Similar to ultrasonography, CT has broad availability, easy execution and high accuracy in diagnosing steatosis. Unfortunately, its application is also limited by suboptimal grading ability for mild-to-moderate steatosis in the clinical setting [87,88]. Additionally, radiation exposure renders CT inappropriate for longitudinal evaluation of NAFLD in early screening and diagnosis, and incidental detection of hepatic steatosis by CT in other indications is common [89]. Therefore, according to current European Association for the Study of the Liver (EASL) clinical practice guidelines, although it is not a primary diagnostic tool, CT remains to be recommended as a routine test of incidental hepatic steatosis.

#### 4.2.3. Controlled Attenuation Parameter (CAP)

CAP, a non-invasive index derived from ultrasound signals, was specifically designed for the detection of hepatic steatosis in individuals with about 10% liver fat without fibrosis or cirrhosis. A previous study showed that CAP can accurately detect and grade steatosis ≥11%, ≥33% and ≥66% with AUROCs of 0.91, 0.95 and 0.89, respectively [90]. Nevertheless, it is worth noting that majority of reports supporting CAP utilized the M-probe for analysis, which has an elevated screening failure rate in obese individuals. In a meta-analysis patient data using the XL-probe, CAP’s accuracy for distinguishing steatosis had a suboptimal performance [91]. CAP levels may increase after eating and its diagnostic performance is heavily associated with the operator’s skills. Besides, there is still no consensus regarding the cutoffs [92]. Despite its limitations, the Asia-Pacific guidelines still recommend CAP as a conventional testing index for the diagnosis and screening of NAFLD because of its low cost and convenience [93].

#### 4.2.4. Magnetic Resonance-Based Techniques

MRI is a non-invasive test that quantitates liver fat amounts with high spatial resolution and no ionizing radiation. MRI has been proposed as an alternative to liver biopsy in clinical NAFLD (e.g., steatohepatitis or fibrosis). With the continuous development of technology, magnetic resonance spectroscopy (MRS), an MRI-based technique, has been refined to accurately detect changes of hepatic steatosis and grade steatosis. Usually, this technique has determined liver fat at 5.56% as a pathological cut-off in a population compared to healthy individuals [94]. MRI-proton density fat fraction (MRI-PDFF) constitutes a more advanced MRI-based diagnostic tool, which can detect liver fat content across the whole liver in an objective, quantitative and reproducible manner [95,96]. Quantitation of steatosis by dividing all the protons in the liver is the main principle of MRI-PDFF. It can be assessed into multiple regions within the liver and has been validated against liver histology. Thus, compared with other imaging tools, MRI-PDFF has resulted in wide applications in NASH trials as an approval endpoint. Currently, MRI-PDFF is superior to CAP in the diagnosis all steatosis stages, with higher accuracy and sensitivity [97]. There are some limitations in MRI-PDFF, including high cost, sophisticated algorithms, and the requirements for MRI instruments and expert operators, which have reduced availability in comparison with ultrasound-based devices. Thus, MRI-PDFF is currently applied only in clinical studies.

## 5. Management of NAFLD

The management of NAFLD and its related diseases comprises diverse tiers, including conservative and surgical treatments. NAFLD treatment is a multimodal endeavor targeting many aspects, including weight loss, lifestyle changes and possible medication optimization.

### 5.1. Lifestyle Modifications and Weight Loss

There is no current specific drug for NAFLD therapy; however, it is widely believed that weight loss is the most critical intervention for obesity and NAFLD [87,98,99]. Moreover, a combination treatment can be beneficial such as lifestyle modifications, increased physical activity and smoking/alcohol cessation [100,101]. In recent years, international guidelines have strongly recommended a target of 7–10% weight loss to be achieved through a healthy structured diet and physical activity [5,102]. The recommended healthy diet that reduces the calorie intake and high-glycemic index foods is supported by a prospective trial [103]. It was shown to reduce the NAFLD activity score, including achieving complete NASH alleviation and fibrosis regression in some patients. Among them, patients losing ≥10% body weight had the greatest benefit, with partial beneficial outcomes observed in patients with weight loss ≥5%. Exercise, even without weight loss, is effective in ameliorating liver fat [104]. A randomized trial demonstrated that supervised exercise programs with moderate aerobic exercise can reduce liver fat compared to counselling [105]. Similarly, high-intensity interval training also shows therapeutic effects on liver fat and whole-body fat mass [106]. In conclusion, there are certain benefits of lifestyle modifications, in that liver-associated mortality is reduced across all BMI levels and the excess risk of liver disease-related death associated with obesity is decreased though reasonable and persistent physical activity.

### 5.2. Pharmacological Treatment

Multiple drugs for limiting NAFLD development and progression have been examined, although none is currently specifically licensed for NAFLD treatment. We summarize currently recommended drugs as shown in Table 2.

#### 5.2.1. Pioglitazone

Pioglitazone, a thiazolidinedione derivative, is a potent activator of the nuclear receptor PPARγ (peroxisome proliferator-activated receptor γ), which is highly expressed in fat tissue, with an important role in adipocyte differentiation as well as in lipid and glucose metabolism [107]. Therefore, pioglitazone ameliorates IR and enhances glucose and lipid metabolism in diabetics [108]. In several PIVENS trials (pioglitazone vs. vitamin E vs. placebo for nondiabetics with NASH), low-dose pioglitazone, vitamin E and placebo were compared, showing that pioglitazone can ameliorate liver enzymes, hepatic steatosis and lobular inflammation, although liver fibrosis was improved [109,110,111,112]. Additionally, a meta-analysis found that the risk of myocardial infarction and cardiovascular death increased after pioglitazone treatment compared with a placebo [113]. Furthermore, Bril et al. showed that although pioglitazone had a substantial anti-steatogenic effect, no further benefit in NAFLD activity score, ballooning or fibrosis occurred [114]. Studies also have demonstrated that besides therapeutic effects there are side effects, including substantial weight gain and lower extremity edema [115]. Despite some safety and tolerability concerns, pioglitazone is presently recommended by EASL in select NAFLD and diabetes cases [102]. A meta-analysis demonstrated pioglitazone is substantially more likely to induce adverse events compared with a placebo in prediabetics or diabetics with NAFLD [112]. In comparison with placebo, pioglitazone had distinct adverse events such as hypoglycemia, chronic lower extremity edema, atypical chest pain or epigastralgia, and back/joint pain. There was no substantial elevation of the odds of pioglitazone discontinuation for serious adverse events versus placebo. Meanwhile, between pioglitazone and placebo, no remarkable difference in the risk of adverse events was found. A total of four deaths were reported in the trial, including two patients each in the pioglitazone and placebo groups.

#### 5.2.2. Vitamin E

Reactive oxygen species synthesis is critical for NASH progression. The efficacy of vitamin E can reduce oxidative stress, and vitamin E has thus been evaluated in NASH cases [116], which is supported by currently available guidelines as potential therapy in select NASH cases [5]. Several clinical trials have demonstrated that vitamin E improves liver function and reduces oxidative stress while not causing a significant reduction in histological grade [117]. One study that compared pioglitazone combined with vitamin E and vitamin E alone showed that the levels of serum ALT decreased in both groups, with a remarkable histological improvement only detected in the combination group [118]. High-dose vitamin E also increased the risk of all-cause mortality [87]; thus, vitamin E utilization should be taken into consideration for possible side-effects, which mainly include a higher risk of bleeding and adverse cardiovascular outcomes. A proof-of-concept, randomized, double-blind, placebo-controlled study performed in 2010–2016 showed that vitamin E alone and placebo are similar in improving the primary liver histological outcome, with reduced efficacy compared with the combined use of vitamin E and pioglitazone [114]. Vitamin E and placebo showed no differences in the primary outcome or the percentage of individuals with improved steatosis, inflammation, ballooning, and/or fibrosis. In addition, four individuals died during the trial of cardiovascular complications, including two each in the vitamin E alone and combination groups, respectively. Overall, orally administered vitamin E was well-tolerated, with no serious adverse events. Compared to placebo, side effects were reported in the combination therapy, such as increased peripheral edema, weight gain and hypoglycemia. Other side effects did not occur in the combination group, including bladder cancer, osteoporosis, and osteoporotic bone fractures.

#### 5.2.3. Glucagon-Like Peptide 1 (GLP-1) Agonists

Since Type 2 diabetes and NAFLD are closely associated, antidiabetic drugs were examined for NAFLD treatment. Endogenous GLP-1 constitutes an incretin hormone synthesized by gut mucosal L cells. GLP-1 agonists show prolonged half-lives compared with endogenous GLP-1 but exert similar effects, which stimulate insulin secretion, inhibit glucagon secretion and reduce liver glucose production [119]. Further, the latter agonists also suppress central appetite and induce weight loss, which are beneficial outcomes in obese NAFLD/NASH patients. In multiple clinical studies, the GLP-1 agonist liraglutide has been shown to be an effective drug for Type 2 diabetes that can yield a good glycemic control and improve liver-enzyme levels [120]. Additionally, liraglutide can decrease hepatic steatosis and increase weight loss, with beneficial effects on the histologic resolution of NASH [119]. Semaglutide represents an additional GLP-1 agonist, with approval for diabetes treatment [120], which is being studied for use in weight management [91]. It was previously shown that semaglutide induces weight loss and improves glycemic control in obese individuals [120] and diabetics [121,122]. Semaglutide has a similar mechanism of action to liraglutide but with enhanced metabolic effects [123,124]. Since diabetes is an important component of NAFLD development and metabolic syndrome, the efficacy, safety and good tolerability make the GLP-1 agonist a suitable treatment tool for NAFLD. In a semaglutide vs. liraglutide trial, there was an increase in clinically meaningful levels of weight loss in the semaglutide group, with substantial improvement in diverse cardiometabolic parameters [125]. In addition, gastrointestinal diseases were the commonest adverse events induced by semaglutide and liraglutide.

#### 5.2.4. Sodium Glucose Cotransporter 2 (SGLT-2) Inhibitors

SGLT-2 suppressors, as the latest group of antidiabetic medications, inhibit kidney glucose reabsorption, which results in prominent glycosuria. At therapeutic levels, glucose (60 to 100 g) is excreted in urine, thereby directly decreasing circulatory glucose amounts and reducing blood glucose [126]. Currently, five oral SGLT2 inhibitors have been approved by the European Medicines Agency and the Federal Drug Administration, including canagliflozin, dapagliflozin, empagliflozin and ertugliflozin. Clinically, all SGLT2 suppressors are utilized as glucose-lowering drugs, with additional benefits of weight loss and blood pressure lowering, similar to GLP-1 agonists [127,128]. Whether all SGLT2 suppressors should be administered depends largely on estimated glomerular filtration rate. The adjustment recommendations of SGLT-2 inhibitors should refer to kidney function and specific indications of agents within the class [127]. There were also some adverse events reported, including polyuria due to diuresis and fungal genitourinary infections [129]. Meta-analyses and observational trials suggested that although the majority of polyuria cases are detected upon initiation, treatment may be discontinued in some individuals [130]. Numerous drugs with diverse mechanisms of action, targeting lipid metabolism, inflammation or fibrosis have been developed for NASH therapy [8,131].

Obeticholic acid, elafibranor, selonsertib and cenicriviroc are presently in phase III randomized controlled studies for assessing their possible therapeutic values in NAFLD [132,133]. Some phase II and III randomized controlled studies have examined the efficacy and safety of these new drugs in NAFLD and NASH [134], and may become more promising new agents.

#### 5.2.5. Selective PPARα Modulator

Selective PPARα modulator (SPPARMα) drugs may selectively control the transcription of PPARα target genes contributing to beneficial outcomes, but not deleterious effects. PPARα controls lipid and lipoprotein metabolism by transcriptionally regulating genes contributing to serum TG reduction and HDL-C elevation [135]. Pemafibrate, one of the novel SPPARMα drugs, was manufactured by Kowa Company for improved efficiency and safety. Therefore, SPPARMα might have an improved benefit–risk balance in comparison with PPARα agonists. To date, clinical studies have shown pemafibrate exerts superior effects in serum TG reduction and HDL-C increase as well as in its safety profile [136].

### 5.3. Promising Drugs in Current Clinical Trials

NAFLD/NASH clinical trials worldwide mainly involve drugs, behavior, diagnosis, dietary supplements, equipment, surgery, herbal medicines and other therapies/methods. Currently, the number of drug-related trials (monotherapy and combination) is the largest from the perspective of intervention, especially in terms of phase II trials (IIa, IIa/IIb, IIb). However, few drugs have entered phase III trials. In addition, two other trials, small-sample randomized controlled trials of marketed drugs in NAFLD/NASH, have entered phase IV clinical trials that mainly investigate their therapeutic potential and the need for initiating clinical trials. The two trials were: (1) monotherapy with evogliptin (completed), initiated by Dong-A Socio Holdings; (2) combination therapy of pioglitazone and empagliflozin (in progress), initiated by Getz Pharma. Considering the number of phase III clinical projects and the status of clinical development, it remains difficult to develop NAFLD/NASH drugs. Obeticholic acid, lanifibranor (IVA337), resmetirom (MGL-3196) and semaglutide are the four candidates in phase III clinical trials that are progressing rapidly as monotherapies. The four candidates have a metabolic mechanism of action and target FXR, PPAR, THR-β and GLP-1R, respectively, with the exception of semaglutide, which is a peptide; the other three candidates are small molecule compounds. Recently, the American Association for the Study of Liver Diseases (AASLD) brings together cutting-edge academic developments in the field of liver disease, which has made remarkable achievements in NAFLD. According to AASLD 2022, Stephen et al. published results for efruxifermin in NASH cases with fibrosis from a randomized, double-blind, placebo-controlled, phase II2b study. Meanwhile, they also reported a 36 week placebo-controlled phase II study, in which PXL065 reduced liver fat amounts and improved liver histology without PPARγ-associated side effects in NASH cases. Additionally, topline data have been published from a new analysis of the REGENERATE trial of obeticholic acid for NASH treatment. The following investigational drugs in NAFLD/NASH are shown in Table 3.

### 5.4. Bariatric Surgery

In the past few years, bariatric surgery has made great progress. Its benefits have been well established concerning weight loss and the improvement of diverse metabolic disorders [137,138]. Bariatric surgery ameliorates liver fat and may even alleviate all histological lesions in NASH, e.g., fibrosis. It is a cost-effective therapeutic option for all obese NASH cases, even in individuals with advanced fibrosis [139]. Compared to conservative treatment, bariatric surgery even markedly improves long-term overall survival [138,140,141]. Currently, bariatric surgery has been utilized to treat metabolic syndrome and diabetes in Asian individuals, and sleeve gastrectomy is considered the most commonly applied operation [142]. Regarding NAFLD-specific outcomes, multiple currently published meta-analyses have not considered bariatric surgery as a therapeutic option. A combination of pharmacological treatment and bariatric surgery may be a better option. A recent meta-analysis indicated that pioglitazone administered in combination with Roux-Y gastric bypass surgery has a better effect on NAFLD activity [143]. It should be also noted that although bariatric surgery solely ameliorates NAFLD and related diseases, its impact on prognosis needs to be considered [144]. After undergoing bariatric surgery, a small number of patients developed NASH or had aggravated liver disease, including decompensation and even liver transplantation [145,146]. Since diverse surgeries have distinct effects on postsurgical physiological remodeling, bariatric surgery requires further investigation as an established option for specifically treating NAFLD.

## 6. Conclusions

The NAFLD epidemic continues unabated, and NAFLD may become one of the most important chronic liver disorders globally in the near future. Despite impressive progress made in the last four decades in understanding the natural history and underlying biology of NAFLD, multiple challenges remain. Currently, NAFLD has not received adequate attention by health care workers and the whole community. This review suggests that there are multiple factors hampering the development of highly effective therapies in the field. A critical diagnostic challenge is continuous reliance on liver biopsy. Reliable biomarkers accurately diagnosing and staging NAFLD across the whole disease spectrum have not been identified [147]. Ideally, a combination of diagnostic and prognostic biomarkers would yield a better benefit in the detection of high-risk cases and in therapeutic effect. Another important challenge of NAFLD is its heterogeneity and complex pathogenesis, which ultimately leads to the current limited understanding of disease phenotypes. Indeed, phenotypes are better targets to permit an appropriate therapeutic choice and accurate prognosis. Current studies on effective therapeutic agents in NAFLD are now concentrated on various potential aspects, e.g., controlling food intake, improving energy consumption, and decreasing hepatic steatosis and preventing its effects on the liver. Once achieved, treatments for NAFLD will be more targeted and individualized.

Taken together, it is increasingly clear that regardless of current or future progress in diagnostic testing and pharmacological treatment, a healthy lifestyle and weight loss remain important in preventive and therapeutic approaches applied for NAFLD.

## Figures and Tables

**Table 1:** Potential biomarkers in NAFLD.

NAFLD Biomarkers	Biomarker Types	Diagnosis Type
Waist circumference, BMI, triglycerides, γ-glutamyl transferase	Serum, panels	NAFL
ALT/AST, BMI, sex, presence of type 2 diabetes,	Serum, panels	NAFL
Triglycerides	Serum	NAFL
Cholesterol	Serum	NAFL
γ-glutamyl transferase	Serum	NAFL
ALT	Serum	NAFL
AST	Serum	NAFL
Insulin, AST, ALT/AST	Serum	Metabolic syndrome, T2D
α2-macroglobulin, haptoglobin, apolipoprotein A1, GGT, total bilirubin, ALT	Serum	NAFL
Basal hemoglobin	Omics-based	NAFL
Bile acids/glutathione	Omics-based	NAFL
Short-chain fatty acids/eicosanoids	Omics-based	NAFL
miR-122, miR-192, miR-16, miR-21, miR-27b, miR-197, miR-34a, miR-375, miR-451, etc.	Noncoding RNA	NAFL
Cytokeratin-18	Serum	NASH/inflammation
Fibroblast growth factor 12, CK-18	Serum	NASH/inflammation
C-reactive protein, TNF, IL-6, IL-1, IL-1RA, CXCL10	Serum, plasma	NASH/inflammation
Activated plasminogen activator inhibitor 1	Plasma	NASH
Hydroxyoctadecadienoic acid 9 and 13, oxo-octadecadienoic acids	Omics-based	NASH
Linoleic acid/hydroxyoctadecadienoic acid 13, age, BMI, AST	Serum, omics-based, panels	NASH
miR-122, miR-192, miR-16, miR-21, miR-27b, miR-197, miR-34a, miR-375, miR-30c, miR-22, IncRNA LeXis, etc.	Noncoding RNA	NASH
AST/ALT, AST/platelet	Serum, plasma	Fibrosis
BMI, AST/ALT, presence of diabetes	Serum, panels	Fibrosis
Age, AST, ALT, platelet count	Serum, panels	Fibrosis
Age, presence of diabetes, BMI, platelets, albumin, AST/ALT	Serum, panels, plasma	Fibrosis
Hyaluronic acid	Serum	Fibrosis, cirrhosis
Procollagen III amino-terminal peptide	Serum	Fibrosis
Pro-C3	Serum	NASH, fibrosis
Metalloprotease-1 inhibitor	Serum	NASH, fibrosis
Laminin	Serum	Fibrosis
Hyaluronic acid, metalloprotease-1 inhibitor, procollagen III amino-terminal peptide	Serum	Fibrosis
Age, sex, gender, bilirubin, γ-glutamyl transferase, hyaluronic acid, α2-macroglobulin	Serum, panels	Fibrosis
miR-122, miR-192, miR-16, miR-21, miR-27b, miR-197, miR-30c, IncRNA APTR, IncRNA RP11-128N14.5, IncRNA GAS5, etc.	Noncoding RNA	Fibrosis

**Table 2:** Potential use of off-label pharmacological treatment.

Drugs	Effects on the Liver	Other Benefits	Key Adverse Events
Pioglitazone	Improves hepatic steatosis, necroinflammation, and fibrosis	Improves insulin resistance and glycemic control	Might improve weight gain, bone loss, and fluid retention
Vitamin E	Improves hepatic steatosis and necroinflammation	Might prevent decompensation and mortality in patients with advanced liver fibrosis	Might increase risk of all-cause mortality, bleeding, heart failure, and hemorrhagic stroke
GLP-1 agonists	Improves hepatic steatosis and necroinflammation	Improves glycemic control; reduces body weight	Increases nausea, vomiting, diarrhea, and constipation
SGLT2 inhibitors	Improves hepatic steatosis, necroinflammation, and liver enzymes	Improves glycemic control; reduces body weight; might have reno-protective benefits	Might have acute kidney injury, euglycemic diabetic ketoacidosis; genitourinary infection, and fractures

**Table 3:** Potential Drugs in NAFLD/NASH under studies.

Drug Names	Molecule Types	Targets	Mechanism	Phase	Status
Pegozafermin (BIO-89-100)	Protein	FGF21	Metabolism	II	In progress
BI 456906	Peptide	GLP-1R /GCGR	Metabolism	II	In progress
HM15211	Peptide	GCGR/GIPR/GLP-1R	Metabolism	II	In progress
Tirzepatide	Peptide	GIPR/GLP-1R	Metabolism	II	In progress
BMS-986263	Nucleotide	HSP47	Anti-fibrosis	II	In progress
ION224	Nucleotide	DGAT2	Metabolism	II	In progress
BFKB8488A	Antibody	FGF21	Metabolism	II	In progress
BI 685509	Unknown	sGC	Metabolism	II	In progress
Chiglitazar sodium	Small molecule	PPAR	Metabolism	II	In progress
Ervogastat (PF-06865571)	Small molecule	DGAT2	Metabolism	II	In progress
HEC96719	Small molecule	FXR	Metabolism	II	In progress
Tipelukast (MN-001)	Small molecule	Multi-target	Anti-inflammatory + anti-fibrosis	II	In progress
Tropifexor	Small molecule	FXR	Metabolism	II	In progress
ZED1227	Small molecule	TG2	Metabolism	II	In progress
Pegbelfermin (BMS-986036)	Protein	FGF21	Metabolism	II	Completed
PXL770	Unknown	AMPK	Metabolism	II	Completed
Licogliflozin (LIK066)	Small molecule	SGLT1/2	Metabolism	II	Completed
PXL065	Small molecule	PPAR	Metabolism	II	Completed
BOS-580	Protein	FGF21	Metabolism	IIa	In progress
Efinopegdutide (MK-6024)	Peptide	GCGR/ GLP-1R	Metabolism	IIa	In progress
FM101	Small molecule	Unknown	Unknown	IIa	In progress
HPG1860	Small molecule	FXR	Metabolism	IIa	In progress
TERN-501	Small molecule	THR-β	Metabolism	IIa	In progress
Epeleuton (DS102)	Small molecule	Unknown	Unknown	IIa	Completed
EYP0001a	Small molecule	FXR	Metabolism	IIa	Completed
TERN-101	Small molecule	FXR	Metabolism	IIa	Completed
ASC41	Small molecule	THR-β	Metabolism	IIa/IIb	Not started
Rencofilstat	Small molecule	Cyclophilin	Anti-fibrosis	IIb	Not started
Efruxiferimin	Protein	FGF21	Metabolism	IIb	In progress
NGM282	Protein	FGF19	Metabolism	IIb	In progress
MK-3655	Antibody	β-Klotho/FGFR1c receptor	Metabolism	IIb	In progress
ASC40 (TVB-2640)	Small molecule	FASN	Metabolism	IIb	In progress
Icosabutate	Small molecule	Lipid regulation	Metabolism	IIb	In progress
VK2809	Small molecule	THR-β	Metabolism	IIb	In progress
Belapectin	Small molecule	Galectin-3	Anti-fibrosis	II/III	In progress
Azemiglitazone (MSDC-0602K)	Small molecule	Mitochondria	Metabolism	III	Not started
Semaglutide	Peptide	GLP-1R	Metabolism	III	In progress
Lanifibranor (IVA337)	Small molecule	PPAR	Metabolism	III	In progress
Obeticholic Acid	Small molecule	FXR	Metabolism	III	In progress
Resmetirom (MGL-3196)	Small molecule	THR-β	Metabolism	III	In progress
Secukinumab	Antibody	IL-17A	Anti-fibrosis	III	Completed
Selonsertib	Small molecule	ASK1	Anti-inflammatory	III	Terminated
Evogliptin	Small molecule	DPP-4	Metabolism	IV	Completed
Dabigatran Etexilate + Rabeprazo I sodium	Small molecule + small molecule	Thrombin+ proton-pump inhibitors	Unknown	I	Completed
NN9499+Semaglutide	Protein + peptide	FGF21+GLP-1R	Metabolism	II	In progress
Semaglutide + Cilofexor + Firsocostat	Peptide + small molecule + small molecule	GLP-1R+FXR+ACC	Metabolism	II	In progress
BI 685509 + Empagliflozin	Unknown	sGC+SGLT-2	Metabolism	II	In progress
Lanifibranor (IVA337) + Empagliflozin	Small molecule + peptide	PPAR+SGLT-2	Metabolism	II	In progress
Tropifexor + Licogliflozin	Small molecule + small molecule	FXR+ SGLT1/2	Metabolism	II	In progress
Semaglutide + Firsocostat	Peptide + small molecule	GLP-1R+FXR	Metabolism	II	Completed
Selonsertib + Cilofexor	Small molecule + small molecule	ASK1+FXR	Anti-inflammatory + metabolism	II	Completed
Firsocostat + Fenofibrate	Small molecule + small molecule	ACC+FXR	Metabolism	II	Completed
Obeticholic Acid + Atorvastatin	Small molecule + small molecule	FXR+HMG-CoA reductase	Metabolism	II	Completed
TERN-501+TERN-101	Small molecule + small molecule	THR-β+FXR	Metabolism	IIa	In progress
Ervogastat (PF-06865574) + Clesacostat (PF-05221304)	Small molecule + small molecule	DGAT2+ACC	Metabolism	IIa	Completed
Pioglitazone + Empagliflozin	Small molecule + small molecule	PPAR+SGLT-2	Metabolism	IV	In progress

## Data Availability

Not applicable.

## References

[1] Younossi Z.M., Corey K.E., Lim J.K. (2021). AGA Clinical Practice Update on Lifestyle Modification Using Diet and Exercise to Achieve Weight Loss in the Management of Nonalcoholic Fatty Liver Disease: Expert Review. Gastroenterology.

[2] Golabi P., Paik J.M., AlQahtani S., Younossi Y., Tuncer G., Younossi Z.M. (2021). Burden of non-alcoholic fatty liver disease in Asia, the Middle East and North Africa: Data from Global Burden of Disease 2009–2019. J. Hepatol..

[3] Lazarus J.V., Mark H.E., Anstee Q.M., Arab J.P., Batterham R.L., Castera L., Cortez-Pinto H., Crespo J., Cusi K., Dirac M.A. (2022). Advancing the global public health agenda for NAFLD: A consensus statement. Nat. Rev. Gastroenterol. Hepatol..

[4] Pouwels S., Sakran N., Graham Y., Leal A., Pintar T., Yang W., Kassir R., Singhal R., Mahawar K., Ramnarain D. (2022). Non-alcoholic fatty liver disease (NAFLD): A review of pathophysiology, clinical management and effects of weight loss. BMC Endocr. Dis..

[5] Chalasani N., Younossi Z., Lavine J.E., Diehl A.M., Brunt E.M., Cusi K., Charlton M., Sanyal A.J. (2012). The Diagnosis and Management of Non-alcoholic Fatty Liver Disease: Practice Guideline by the American Gastroenterological Association, American Association for the Study of Liver Diseases, and American College of Gastroenterology. Gastroenterology.

[6] Powell E.E., Wong V.W.S., Rinella M. (2021). Non-alcoholic fatty liver disease. Lancet.

[7] Paik J.M., Golabi P., Younossi Y., Mishra A., Younossi Z.M. (2020). Changes in the Global Burden of Chronic Liver Diseases From 2012 to 2017: The Growing Impact of NAFLD. Hepatology.

[8] Neuschwander-Tetri B.A. (2020). Therapeutic Landscape for NAFLD in 2020. Gastroenterology.

[9] Donnelly K.L., Smith C.I., Schwarzenberg S.J., Jessurun J., Boldt M.D., Parks E.J. (2005). Sources of fatty acids stored in liver and secreted via lipoproteins in patients with nonalcoholic fatty liver disease. J. Clin. Investig..

[10] Byrne C.D., Targher G. (2015). NAFLD: A multisystem disease. J. Hepatol..

[11] Loomba R., Friedman S.L., Shulman G.I. (2021). Mechanisms and disease consequences of nonalcoholic fatty liver disease. Cell.

[12] Tilg H., Adolph T.E., Dudek M., Knolle P. (2021). Non-alcoholic fatty liver disease: The interplay between metabolism, microbes and immunity. Nat. Metab..

[13] Kumar S., Duan Q., Wu R., Harris E.N., Su Q. (2021). Pathophysiological communication between hepatocytes and non-parenchymal cells in liver injury from NAFLD to liver fibrosis. Adv. Drug Del. Rev..

[14] Yazici D., Sezer H. (2017). Insulin Resistance, Obesity and Lipotoxicity. Obesity and Lipotoxicity.

[15] Eslam M., Sanyal A.J., George J., Int Consensus P. (2020). MAFLD: A Consensus-Driven Proposed Nomenclature for Metabolic Associated Fatty Liver Disease. Gastroenterology.

[16] Eslam M., Newsome P.N., Sarin S.K., Anstee Q.M., Targher G., Romero-Gomez M., Zelber-Sagi S., Wong V.W.S., Dufour J.F., Schattenberg J.M. (2020). A new definition for metabolic dysfunction-associated fatty liver disease: An international expert consensus statement. J. Hepatol..

[17] Fouad Y., Waked I., Bollipo S., Gomaa A., Ajlouni Y., Attia D. (2020). What’s in a name? Renaming ‘NAFLD’ to ‘MAFLD’. Liver Int..

[18] Fouad Y., Elwakil R., Elsahhar M., Said E., Bazeed S., Gomaa A.A., Hashim A., Kamal E., Mehrez M., Attia D. (2021). The NAFLD-MAFLD debate: Eminence vs evidence. Liver Int..

[19] Yu F., Wang Z., Zhang T., Chen X., Xu H., Wang F., Guo L., Chen M., Liu K., Wu B. (2021). Deficiency of intestinal Bmal1 prevents obesity induced by high-fat feeding. Nat. Commun..

[20] Verna E.C., Berk P.D. (2008). Role of Fatty Acids in the Pathogenesis of Obesity and Fatty Liver: Impact of Bariatric Surgery. Semin. Liver Dis..

[21] Postic C., Girard J. (2008). The role of the lipogenic pathway in the development of hepatic steatosis. Diabetes Metab..

[22] Rolo A.P., Teodoro J.S., Palmeira C.M. (2012). Role of oxidative stress in the pathogenesis of nonalcoholic steatohepatitis. Free Radic. Biol. Med..

[23] Zambo V., Simon-Szabo L., Szelenyi P., Kereszturi E., Banhegyi G., Csala M. (2013). Lipotoxicity in the liver. World J. Hepatol..

[24] Samuel V.T., Shulman G.I. (2018). Nonalcoholic Fatty Liver Disease as a Nexus of Metabolic and Hepatic Diseases. Cell Metab..

[25] Eguchi A., Iwasa M. (2021). The Role of Elevated Liver-Type Fatty Acid-Binding Proteins in Liver Diseases. Pharm. Res..

[26] Chmurzynska A. (2006). The multigene family of fatty acid-binding proteins (FABPs): Function, structure and polymorphism. J. Appl. Genet..

[27] Wang Y., Yu W., Li S., Guo D., He J., Wang Y. (2022). Acetyl-CoA Carboxylases and Diseases. Front. Oncol..

[28] Okumura T. (2011). Role of lipid droplet proteins in liver steatosis. J. Physiol. Biochem..

[29] Jeong S.H., Kim H.B., Kim M.C., Lee J.M., Lee J.H., Kim J.H., Kim J.W., Park W.Y., Kim S.Y., Kim J.B. (2018). Hippo-mediated suppression of IRS2/AKT signaling prevents hepatic steatosis and liver cancer. J. Clin. Investig..

[30] Shimomura I., Matsuda M., Hammer R.E., Bashmakov Y., Brown M.S., Goldstein J.L. (2000). Decreased IRS-2 and increased SREBP-1c lead to mixed insulin resistance and sensitivity in livers of lipodystrophic and ob/ob mice. Mol. Cell.

[31] Argo C.K., Northup P.G., Al-Osaimi A.M.S., Caldwell S.H. (2009). Systematic review of risk factors for fibrosis progression in non-alcoholic steatohepatitis. J. Hepatol..

[32] Hagstrom H., Nasr P., Ekstedt M., Hammar U., Stal P., Hultcrantz R., Kechagias S. (2017). Fibrosis stage but not NASH predicts mortality and time to development of severe liver disease in biopsy-proven NAFLD. J. Hepatol..

[33] D’Amico G., Morabito A., D’Amico M., Pasta L., Malizia G., Rebora P., Valsecchi M.G. (2018). New concepts on the clinical course and stratification of compensated and decompensated cirrhosis. Hepatol. Int..

[34] Kawano Y., Cohen D.E. (2013). Mechanisms of hepatic triglyceride accumulation in non-alcoholic fatty liver disease. J. Gastroenterol..

[35] Tilg H., Moschen A.R. (2010). Evolution of Inflammation in Nonalcoholic Fatty Liver Disease: The Multiple Parallel Hits Hypothesis. Hepatology.

[36] Mansouri A., Gattolliat C.-H., Asselah T. (2018). Mitochondrial Dysfunction and Signaling in Chronic Liver Diseases. Gastroenterology.

[37] Berlanga A., Guiu-Jurado E., Porras J.A., Auguet T. (2014). Molecular pathways in non-alcoholic fatty liver disease. Clin. Exp. Gastroenterol..

[38] Engin A. (2017). Non-Alcoholic Fatty Liver Disease. Obes. Lipotoxic..

[39] Sakurai Y., Kubota N., Yamauchi T., Kadowaki T. (2021). Role of Insulin Resistance in MAFLD. Int. J. Mol. Sci..

[40] Jornayvaz F.R., Birkenfeld A.L., Jurczak M.J., Kanda S., Guigni B.A., Jiang D.C., Zhang D., Lee H.-Y., Samuel V.T., Shulman G.I. (2011). Hepatic insulin resistance in mice with hepatic overexpression of diacylglycerol acyltransferase 2. Proc. Natl. Acad. Sci. USA.

[41] Choi C.S., Savage D.B., Kulkarni A., Yu X.X., Liu Z.-X., Morino K., Kim S., Distefano A., Samuel V.T., Neschen S. (2007). Suppression of diacylglycerol acyltransferase-2 (DGAT2), but not DGAT1, with antisense oligonucleotides reverses diet-induced hepatic steatosis and insulin resistance. J. Biol. Chem..

[42] Sajan M.P., Ivey R.A., Lee M.C., Farese R.V. (2015). Hepatic insulin resistance in ob/ob mice involves increases in ceramide, aPKC activity, and selective impairment of Akt-dependent FoxO1 phosphorylation. J. Lipid Res..

[43] Sajan M.P., Lee M.C., Foufelle F., Sajan J., Cleland C., Farese R.V. (2018). Coordinated regulation of hepatic FoxO1, PGC-1 alpha and SREBP-1c facilitates insulin action and resistance. Cell. Signal..

[44] Akazawa Y., Cazanave S., Mott J.L., Elmi N., Bronk S.F., Kohno S., Charlton M.R., Gores G.J. (2010). Palmitoleate attenuates palmitate-induced Bim and PUMA up-regulation and hepatocyte lipoapoptosis. J. Hepatol..

[45] Leveille M., Estall J.L. (2019). Mitochondrial Dysfunction in the Transition from NASH to HCC. Metabolites.

[46] Liu R.Z., Choi W.S., Jain S., Dinakaran D., Xu X., Han W.H., Yang X.H., Glubrecht D.D., Moore R.B., Lemieux H. (2020). The FABP12/PPAR gamma pathway promotes metastatic transformation by inducing epithelial-to-mesenchymal transition and lipid-derived energy production in prostate cancer cells. Mol. Oncol..

[47] Gonzalez-Franquesa A., Patti M.-E. (2017). Insulin Resistance and Mitochondrial Dysfunction. Mitochondrial Dynamics in Cardiovascular Medicine.

[48] Zhang D., Liu Z.X., Choi C.S., Tian L., Kibbey R., Dong J., Cline G.W., Wood P.A., Shulman G.I. (2007). Mitochondrial dysfunction due to long-chain Acyl-CoA dehydrogenase deficiency causes hepatic steatosis and hepatic insulin resistance. Proc. Natl. Acad. Sci. USA.

[49] Schuppan D., Surabattula R., Wang X.Y. (2018). Determinants of fibrosis progression and regression in NASH. J. Hepatol..

[50] Barreyro F.J., Kobayashi S., Bronk S.F., Werneburg N.W., Malhi H., Gores G.J. (2007). Transcriptional regulation of Bim by FoxO3A mediates hepatocyte lipoapoptosis. J. Biol. Chem..

[51] Camps J., Joven J. (2015). Chemokine ligand 2 and paraoxonase-1 in non-alcoholic fatty liver disease: The search for alternative causative factors. World J. Gastroenterol..

[52] Magee N., Zou A., Zhang Y. (2016). Pathogenesis of Nonalcoholic Steatohepatitis: Interactions between Liver Parenchymal and Nonparenchymal Cells. Biomed. Res. Int..

[53] Ikura Y., Ohsawa M., Suekane T., Fukushima H., Itabe H., Jomura H., Nishiguchi S., Inoue T., Naruko T., Ehara S. (2006). Localization of oxidized phosphatidylcholine in nonalcoholic fatty liver disease: Impact on disease progression. Hepatology.

[54] Zhao G.-N., Zhang P., Gong J., Zhang X.-J., Wang P.-X., Yin M., Jiang Z., Shen L.-J., Ji Y.-X., Tong J. (2017). Tmbim1 is a multivesicular body regulator that protects against non-alcoholic fatty liver disease in mice and monkeys by targeting the lysosomal degradation of Tlr4. Nat. Med..

[55] Krenkel O., Tacke F. (2017). Liver macrophages in tissue homeostasis and disease. Nat. Rev. Immun..

[56] Filali-Mouncef Y., Hunter C., Roccio F., Zagkou S., Dupont N., Primard C., Proikas-Cezanne T., Reggiori F. (2022). The menage a trois of autophagy, lipid droplets and liver disease. Autophagy.

[57] Harjumaki R., Pridgeon C.S., Ingelman-Sundberg M. (2021). CYP2E1 in Alcoholic and Non-Alcoholic Liver Injury. Roles of ROS, Reactive Intermediates and Lipid Overload. Int. J. Mol. Sci..

[58] Chung H.K., Kim Y.K., Park J.-H., Ryu M.J., Chang J.Y., Hwang J.H., Lee C.-H., Kim S.-H., Kim H.J., Kweon G.R. (2015). The indole derivative NecroX-7 improves nonalcoholic steatohepatitis in ob/ob mice through suppression of mitochondrial ROS/RNS and inflammation. Liver Int..

[59] Biczo G., Vegh E.T., Shalbueva N., Mareninova O.A., Elperin J., Lotshaw E., Gretler S., Lugea A., Malla S.R., Dawson D. (2018). Mitochondrial Dysfunction, Through Impaired Autophagy, Leads to Endoplasmic Reticulum Stress, Deregulated Lipid Metabolism, and Pancreatitis in Animal Models. Gastroenterology.

[60] Bedogni G., Bellentani S., Miglioli L., Masutti F., Passalacqua M., Castiglione A., Tiribelli C. (2006). The Fatty Liver Index: A simple and accurate predictor of hepatic steatosis in the general population. BMC Gastroenterol..

[61] Kotronen A., Peltonen M., Hakkarainen A., Sevastianova K., Bergholm R., Johansson L.M., Lundbom N., Rissanen A., Ridderstrale M., Groop L. (2009). Prediction of Non-Alcoholic Fatty Liver Disease and Liver Fat Using Metabolic and Genetic Factors. Gastroenterology.

[62] Kahn H.S. (2005). The “lipid accumulation product” performs better than the body mass index for recognizing cardiovascular risk: A population-based comparison. BMC Cardiovasc. Disord..

[63] Dai H., Wang W., Chen R., Chen Z., Lu Y., Yuan H. (2017). Lipid accumulation product is a powerful tool to predict non-alcoholic fatty liver disease in Chinese adults. Nutr. Metab..

[64] Tola E.N., Yalcin S.E., Dugan N. (2017). The predictive effect of inflammatory markers and lipid accumulation product index on clinical symptoms associated with polycystic ovary syndrome in nonobese adolescents and younger aged women. Eur. J. Obstet. Gyn. Reprod. Biol..

[65] Ozcabi B., Demirhan S., Akyol M., Ozturkmen Akay H., Guven A. (2019). Lipid accumulation product is a predictor of nonalcoholic fatty liver disease in childhood obesity. Korean J. Pediatr..

[66] Bedogni G., Kahn H.S., Bellentani S., Tiribelli C. (2010). A simple index of lipid overaccumulation is a good marker of liver steatosis. BMC Gastroenterol..

[67] Fedchuk L., Nascimbeni F., Pais R., Charlotte F., Housset C., Ratziu V., Grp L.S. (2014). Performance and limitations of steatosis biomarkers in patients with nonalcoholic fatty liver disease. Aliment. Pharmacol. Ther..

[68] Jung T.Y., Kim M.S., Hong H.P., Kang K.A., Jun D.W. (2020). Comparative Assessment and External Validation of Hepatic Steatosis Formulae in a Community-Based Setting. J. Clin. Med..

[69] Foschi F.G., Conti F., Domenicali M., Giacomoni P., Borghi A., Bevilacqua V., Napoli L., Berardinelli D., Altini M., Cucchetti A. (2021). External Validation of Surrogate Indices of Fatty Liver in the General Population: The Bagnacavallo Study. J. Clin. Med..

[70] Adams J.C., Lawler J. (2011). The Thrombospondins. Cold Spring Harb. Perspect. Biol..

[71] Lee C.H., Seto W.K., Lui D.T.W., Fong C.H.Y., Wan H.Y., Cheung C.Y.Y., Chow W.S., Woo Y.C., Yuen M.F., Xu A. (2021). Circulating Thrombospondin-2 as a Novel Fibrosis Biomarker of Nonalcoholic Fatty Liver Disease in Type 2 Diabetes. Diabetes Care.

[72] Kimura T., Tanaka N., Fujimori N., Yamazaki T., Katsuyama T., Iwashita Y., Pham J., Joshita S., Pydi S.P., Umemura T. (2021). Serum thrombospondin 2 is a novel predictor for the severity in the patients with NAFLD. Liver Int..

[73] Kozumi K., Kodama T., Murai H., Sakane S., Govaere O., Cockell S., Motooka D., Kakita N., Yamada Y., Kondo Y. (2021). Transcriptomics Identify Thrombospondin-2 as a Biomarker for NASH and Advanced Liver Fibrosis. Hepatology.

[74] Feldstein A.E., Lopez R., Tamimi T.A.R., Yerian L., Chung Y.M., Berk M., Zhang R., McIntyre T.M., Hazen S.L. (2010). Mass spectrometric profiling of oxidized lipid products in human nonalcoholic fatty liver disease and nonalcoholic steatohepatitis. J. Lipid Res..

[75] Liu X.L., Pan Q., Zhang R.N., Shen F., Yan S.Y., Sun C., Xu Z.J., Chen Y.W., Fan J.G. (2016). Disease-specific miR-34a as diagnostic marker of non-alcoholic steatohepatitis in a Chinese population. World J. Gastroenterol..

[76] Pirola C.J., Fernandez Gianotti T., Castano G.O., Mallardi P., San Martino J., Lopez Ledesma M.M.G., Flichman D., Mirshahi F., Sanyal A.J., Sookoian S. (2015). Circulating microRNA signature in non-alcoholic fatty liver disease: From serum non-coding RNAs to liver histology and disease pathogenesis. Gut.

[77] Hernaez R., Lazo M., Bonekamp S., Kamel I., Brancati F.L., Guallar E., Clark J.M. (2011). Diagnostic Accuracy and Reliability of Ultrasonography for the Detection of Fatty Liver: A Meta-Analysis. Hepatology.

[78] Hamaguchi M., Kojima T., Itoh Y., Harano Y., Fujii K., Nakajima T., Kato T., Takeda N., Okuda J., Ida K. (2007). The severity of ultrasonographic findings in nonalcoholic fatty liver disease reflects the metabolic syndrome and visceral fat accumulation. Am. J. Gastroenterol..

[79] Bril F., Ortiz-Lopez C., Lomonaco R., Orsak B., Freckleton M., Chintapalli K., Hardies J., Lai S., Solano F., Tio F. (2015). Clinical value of liver ultrasound for the diagnosis of nonalcoholic fatty liver disease in overweight and obese patients. Liver Int..

[80] Xiao G., Zhu S., Xiao X., Yan L., Yang J., Wu G. (2017). Comparison of Laboratory Tests, Ultrasound, or Magnetic Resonance Elastography to Detect Fibrosis in Patients with Nonalcoholic Fatty Liver Disease: A Meta-Analysis. Hepatology.

[81] Ballestri S., Nascimbeni F., Baldelli E., Marrazzo A., Romagnoli D., Targher G., Lonardo A. (2017). Ultrasonographic fatty liver indicator detects mild steatosis and correlates with metabolic/histological parameters in various liver diseases. Metabolism.

[82] Nelson S.M., Hoskins J.D., Lisanti C., Chaudhuri J. (2020). Ultrasound Fatty Liver Indicator: A Simple Tool for Differentiating Steatosis from Nonalcoholic Steatohepatitis: Validity in the Average Obese Population. J. Ultrasound Med..

[83] Xavier S.A., Monteiro S.O., Arieira C.M., Castro F.D., Magalhaes J.T., Leite S.M., Marinho C.M., Cotter J.B. (2021). US-FLI score—Is it possible to predict the steatosis grade with an ultrasonographic score?. Mol. Genet. Metab..

[84] Byra M., Han A., Boehringer A.S., Zhang Y.N., O′Brien W.D., Erdman J.W., Loomba R., Sirlin C.B., Andre M. (2022). Liver Fat Assessment in Multiview Sonography Using Transfer Learning with Convolutional Neural Networks. J. Ultrasound Med..

[85] Han A., Byra M., Heba E., Andre M.P., Erdman J.W., Loomba R., Sirlin C.B., O′Brien W.D. (2020). Noninvasive Diagnosis of Nonalcoholic Fatty Liver Disease and Quantification of Liver Fat with Radiofrequency Ultrasound Data Using One-dimensional Convolutional Neural Networks. Radiology.

[86] Sanabria S.J., Pirmoazen A.M., Dahl J., Kamaya A., El Kaffas A. (2022). Comparative Study of Raw Ultrasound Data Representations in Deep Learning to Classify Hepatic Steatosis. Ultrasound Med. Biol..

[87] Jennison E., Patel J., Scorletti E., Byrne C.D. (2019). Diagnosis and management of non-alcoholic fatty liver disease. Postgrad. Med. J..

[88] Lee S.S., Park S.H., Kim H.J., Kim S.Y., Kim M.Y., Kim D.Y., Suh D.J., Kim K.M., Bae M.H., Lee J.Y. (2010). Non-invasive assessment of hepatic steatosis: Prospective comparison of the accuracy of imaging examinations. J. Hepatol..

[89] Kontrick A.V., VanWagner L.B., Yeh C., Courtney D.M. (2021). Hepatic Steatosis: An Incidental Finding That Deserves Attention. Acad. Emerg. Med..

[90] Sasso M., Beaugrand M., de Ledinghen V., Douvin C., Marcellin P., Poupon R., Sandrin L., Miette V. (2010). Controlled attenuation parameter (cap): A novel vcte (tm) guided ultrasonic attenuation measurement for the evaluation of hepatic steatosis: Preliminary study and validation in a cohort of patients with chronic liver disease from various causes. Ultrasound Med. Biol..

[91] Siddiqui M.S., Vuppalanchi R., Van Natta M.L., Hallinan E., Kowdley K.V., Abdelmalek M., Neuschwander-Tetri B.A., Loomba R., Dasarathy S., Brandman D. (2019). Vibration-Controlled Transient Elastography to Assess Fibrosis and Steatosis in Patients with Nonalcoholic Fatty Liver Disease. Clin. Gastroenterol. Hepatol..

[92] Petroff D., Blank V., Newsome P.N., Shalimar, Voican C.S., Thiele M., de Ledinghen V., Baumeler S., Chan W.K., Perlemuter G. (2021). Assessment of hepatic steatosis by controlled attenuation parameter using the M and XL probes: An individual patient data meta-analysis. Lancet Gastroenterol. Hepatol..

[93] Wong V.W.S., Chan W.-K., Chitturi S., Chawla Y., Dan Y.Y., Duseja A., Fan J., Goh K.L., Hamaguchi M., Hashimoto E. (2018). Asia-Pacific Working Party on Non-alcoholic Fatty Liver Disease guidelines 2017Part 1: Definition, risk factors and assessment. J. Gastroenterol. Hepatol..

[94] Szczepaniak L.S., Nurenberg P., Leonard D., Browning J.D., Reingold J.S., Grundy S., Hobbs H.H., Dobbins R.L. (2005). Magnetic resonance spectroscopy to measure hepatic triglyceride content: Prevalence of hepatic steatosis in the general population. Am. J. Physiol. Endocrinol. Metab..

[95] Piazzolla V.A., Mangia A. (2020). Noninvasive Diagnosis of NAFLD and NASH. Cells.

[96] Loomba R., Neuschwander-Tetri B.A., Sanyal A., Chalasani N., Diehl A.M., Terrault N., Kowdley K., Dasarathy S., Kleiner D., Behling C. (2020). Multicenter Validation of Association Between Decline in MRI-PDFF and Histologic Response in NASH. Hepatology.

[97] Bae J.S., Lee D.H., Suh K.S., Kim H., Lee K.B., Lee J.Y., Han J.K. (2022). Noninvasive assessment of hepatic steatosis using a pathologic reference standard: Comparison of CT, MRI, and US-based techniques. Ultrasonography.

[98] Fan J.-G., Kim S.-U., Wong V.W.-S. (2017). New trends on obesity and NAFLD in Asia. J. Hepatol..

[99] Hydes T.J., Summers N., Brown E., Alam U., Thomaides-Brears H., Wilding J.P.H., Cuthbertson D.J. (2020). Mechanisms, screening modalities and treatment options for individuals with non-alcoholic fatty liver disease and type 2 diabetes. Diabet. Med..

[100] Zhang C., Yang M. (2021). Current Options and Future Directions for NAFLD and NASH Treatment. Int. J. Mol. Sci..

[101] Trauner M., Fuchs C.D. (2022). Novel therapeutic targets for cholestatic and fatty liver disease. Gut.

[102] Marchesini G., Day C.P., Dufour J.F., Canbay A., Nobili V., Ratziu V., Tilg H., Roden M., Gastaldelli A., Yki-Jaevinen H. (2016). EASL-EASD-EASO Clinical Practice Guidelines for the management of non-alcoholic fatty liver disease. J. Hepatol..

[103] Thomsen M.N., Skytte M.J., Samkani A., Carl M.H., Weber P., Astrup A., Chabanova E., Fenger M., Frystyk J., Hartmann B. (2022). Dietary carbohydrate restriction augments weight loss-induced improvements in glycaemic control and liver fat in individuals with type 2 diabetes: A randomised controlled trial. Diabetologia.

[104] Hallsworth K., Thoma C., Hollingsworth K.G., Cassidy S., Anstee Q.M., Day C.P., Trenell M.I. (2015). Modified high-intensity interval training reduces liver fat and improves cardiac function in non-alcoholic fatty liver disease: A randomized controlled trial. Clin. Sci..

[105] Cuthbertson D.J., Shojaee-Moradie F., Sprung V.S., Jones H., Pugh C.J.A., Richardson P., Kemp G.J., Barrett M., Jackson N.C., Thomas E.L. (2016). Dissociation between exercise-induced reduction in liver fat and changes in hepatic and peripheral glucose homoeostasis in obese patients with non-alcoholic fatty liver disease. Clin. Sci..

[106] Sabag A., Barr L., Armour M., Armstrong A., Baker C.J., Twigg S.M., Chang D., Hackett D.A., Keating S.E., George J. (2022). The Effect of High-intensity Interval Training vs Moderate-intensity Continuous Training on Liver Fat: A Systematic Review and Meta-Analysis. J. Clin. Endocrinol. Metab..

[107] Gross B., Pawlak M., Lefebvre P., Staels B. (2017). PPARs in obesity-induced T2DM, dyslipidaemia and NAFLD. Nat. Rev. Endocrinol..

[108] Le P., Chaitoff A., Rothberg M.B., McCullough A., Alkhouri N. (2020). Trends in pioglitazone use among US adults with type 2 diabetes and suspected nonalcoholic fatty liver disease. Expert Opin. Investig. Drug..

[109] Brunt E.M., Kleiner D.E., Wilson L.A., Sanyal A.J., Neuschwander-Tetri B.A., Nonalcoholic Steatohepatitis Clinical Research Network (2019). Improvements in Histologic Features and Diagnosis Associated with Improvement in Fibrosis in Nonalcoholic Steatohepatitis: Results from the Nonalcoholic Steatohepatitis Clinical Research Network Treatment Trials. Hepatology.

[110] Gawrieh S., Wilson L.A., Yates K.P., Cummings O.W., Vilar-Gomez E., Ajmera V., Kowdley K.V., Rosenberg W.M., Tonascia J., Chalasani N. (2021). Relationship of ELF and PIIINP with Liver Histology and Response to Vitamin E or Pioglitazone in the PIVENS Trial. Hepatol. Commun..

[111] Corey K.E., Wilson L.A., Altinbas A., Yates K.P., Kleiner D.E., Chung R.T., Krauss R.M., Chalasani N., Bringman D., Dasarathy S. (2019). Relationship between resolution of non-alcoholic steatohepatitis and changes in lipoprotein sub-fractions: A post-hoc analysis of the PIVENS trial. Aliment. Pharmacol. Ther..

[112] Lian J., Fu J. (2021). Pioglitazone for NAFLD Patients with Prediabetes or Type 2 Diabetes Mellitus: A Meta-Analysis. Front. Endocrinol..

[113] Nissen S.E., Wolski K. (2007). Effect of rosiglitazone on the risk of myocardial infarction and death from cardiovascular causes. N. Engl. J. Med..

[114] Bril F., Biernacki D.M., Kalavalapalli S., Lomonaco R., Subbarayan S.K., Lai J., Tio F., Suman A., Orsak B.K., Hecht J. (2019). Role of Vitamin E for Nonalcoholic Steatohepatitis in Patients with Type 2 Diabetes: A Randomized Controlled Trial. Diabetes Care.

[115] Ratziu V., Giral P., Jacqueminet S., Charlotte F., Hartemann-Heurtier A., Serfaty L., Podevin P., Lacorte J.-M., Bernhardt C., Bruckert E. (2008). Rosiglitazone for nonalcoholic steatohepatitis: One-year results of the randomized placebo-controlled fatty liver improvement with rosiglitazone therapy (FLIRT) trial. Gastroenterology.

[116] Dong J., Viswanathan S., Adami E., Singh B.K., Chothani S.P., Ng B., Lim W.W., Zhou J., Tripathi M., Ko N.S.J. (2021). Hepatocyte-specific IL11 cis-signaling drives lipotoxicity and underlies the transition from NAFLD to NASH. Nat. Commun..

[117] Gurka M.J., Mack J.A., Chi X., DeBoer M.D. (2021). Use of metabolic syndrome severity to assess treatment with vitamin E and pioglitazone for non-alcoholic steatohepatitis. J. Gastroenterol. Hepatol..

[118] Singh S., Osna N.A., Kharbanda K.K. (2017). Treatment options for alcoholic and non-alcoholic fatty liver disease: A review. World J. Gastroenterol..

[119] Armstrong M.J., Gaunt P., Aithal G.P., Barton D., Hull D., Parker R., Hazlehurst J.M., Guo K., Abouda G., Aldersley M.A. (2016). Liraglutide safety and efficacy in patients with non-alcoholic steatohepatitis (LEAN): A multicentre, double-blind, randomised, placebo-controlled phase 2 study. Lancet.

[120] Cusi K. (2019). Incretin-Based Therapies for the Management of Nonalcoholic Fatty Liver Disease in Patients with Type 2 Diabetes. Hepatology.

[121] Newsome P.N., Buchholtz K., Cusi K., Linder M., Okanoue T., Ratziu V., Sanyal A.J., Sejling A.S., Harrison S.A., NN9931-4296 Investigators (2021). A Placebo-Controlled Trial of Subcutaneous Semaglutide in Nonalcoholic Steatohepatitis. N. Engl. J. Med..

[122] Kushner R.F., Calanna S., Davies M., Dicker D., Garvey W.T., Goldman B., Lingvay I., Thomsen M., Wadden T.A., Wharton S. (2020). Semaglutide 2.4 mg for the Treatment of Obesity: Key Elements of the STEP Trials 1 to 5. Obesity.

[123] Aroda V.R., Ahmann A., Cariou B., Chow F., Davies M.J., Jodar E., Mehta R., Woo V., Lingvay I. (2019). Comparative efficacy, safety, and cardiovascular outcomes with once-weekly subcutaneous semaglutide in the treatment of type 2 diabetes: Insights from the SUSTAIN 1-7 trials. Diabetes Metab..

[124] Husain M., Birkenfeld A.L., Donsmark M., Dungan K., Eliaschewitz F.G., Franco D.R., Jeppesen O.K., Lingvay I., Mosenzon O., Pedersen S.D. (2019). Oral Semaglutide and Cardiovascular Outcomes in Patients with Type 2 Diabetes. N. Engl. J. Med..

[125] Rubino D.M., Greenway F.L., Khalid U., O’Neil P.M., Rosenstock J., Sorrig R., Wadden T.A., Wizert A., Garvey W.T., STEP 8 Investigators (2022). Effect of Weekly Subcutaneous Semaglutide vs Daily Liraglutide on Body Weight in Adults with Overweight or Obesity Without Diabetes the STEP 8 Randomized Clinical Trial. JAMA J. Am. Med. Assoc..

[126] O’Neil P.M., Birkenfeld A.L., McGowan B., Mosenzon O., Pedersen S.D., Wharton S., Carson C.G., Jepsen C.H., Kabisch M., Wilding J.P.H. (2018). Efficacy and safety of semaglutide compared with liraglutide and placebo for weight loss in patients with obesity: A randomised, double-blind, placebo and active controlled, dose-ranging, phase 2 trial. Lancet.

[127] Nauck M.A., Meier J.J. (2019). Are all GLP-1 agonists equal in the treatment of type 2 diabetes?. Eur. J. Endocrinol..

[128] Brown E., Rajeev S.P., Cuthbertson D.J., Wilding J.P.H. (2019). A review of the mechanism of action, metabolic profile and haemodynamic effects of sodium-glucose co-transporter-2 inhibitors. Diabetes Obes. Metab..

[129] Brown E., Heerspink H.J.L., Cuthbertson D.J., Wilding J.P.H. (2021). SGLT2 inhibitors and GLP-1 receptor agonists: Established and emerging indications. Lancet.

[130] Hussein H., Zaccardi F., Khunti K., Davies M.J., Patsko E., Dhalwani N.N., Kloecker D.E., Ioannidou E., Gray L.J. (2020). Efficacy and tolerability of sodium-glucose co-transporter-2 inhibitors and glucagon-like peptide-1 receptor agonists: A systematic review and network meta-analysis. Diabetes Obes. Metab..

[131] Li D., Wang T., Shen S., Fang Z., Dong Y., Tang H. (2017). Urinary tract and genital infections in patients with type 2 diabetes treated with sodium-glucose co-transporter 2 inhibitors: A meta-analysis of randomized controlled trials. Diabetes Obes. Metab..

[132] Younossi Z.M., Ratziu V., Loomba R., Rinella M., Anstee Q.M., Goodman Z., Bedossa P., Geier A., Beckebaum S., Newsome P.N. (2019). Obeticholic acid for the treatment of non-alcoholic steatohepatitis: Interim analysis from a multicentre, randomised, placebo-controlled phase 3 trial. Lancet.

[133] Yamashita S., Masuda D., Matsuzawa Y. (2020). Pemafibrate, a New Selective PPAR alpha Modulator: Drug Concept and Its Clinical Applications for Dyslipidemia and Metabolic Diseases. Curr. Atheroscler. Rep..

[134] Kinoshita M., Yokote K., Arai H., Iida M., Ishigaki Y., Ishibashi S., Umemoto S., Egusa G., Ohmura H., Okamura T. (2018). Japan Atherosclerosis Society (JAS) Guidelines for Prevention of Atherosclerotic Cardiovascular Diseases 2017. J. Atheroscler. Thromb..

[135] Zhang D.Y., Zhu L., Liu H.N., Tseng Y.J., Weng S.Q., Liu T.T., Dong L., Shen X.Z. (2019). The protective effect and mechanism of the FXR agonist obeticholic acid via targeting gut microbiota in non-alcoholic fatty liver disease. Drug Des. Dev. Ther..

[136] Targher G., Byrne C.D., Tilg H. (2020). NAFLD and increased risk of cardiovascular disease: Clinical associations, pathophysiological mechanisms and pharmacological implications. Gut.

[137] O’Brien P.E., Hindle A., Brennan L., Skinner S., Burton P., Smith A., Crosthwaite G., Brown W. (2019). Long-Term Outcomes After Bariatric Surgery: A Systematic Review and Meta-analysis of Weight Loss at 10 or More Years for All Bariatric Procedures and a Single-Centre Review of 20-Year Outcomes After Adjustable Gastric Banding. Obes. Surg..

[138] Aminian A., Wilson R., Al-Kurd A., Tu C., Milinovich A., Kroh M., Rosenthal R.J., Brethauer S.A., Schauer P.R., Kattan M.W. (2022). Association of Bariatric Surgery with Cancer Risk and Mortality in Adults with Obesity. JAMA J. Am. Med. Assoc..

[139] Lassailly G., Caiazzo R., Ntandja-Wandji L.C., Gnemmi V., Baud G., Verkindt H., Ningarhari M., Louvet A., Leteurtre E., Raverdy V. (2020). Bariatric Surgery Provides Long-term Resolution of Nonalcoholic Steatohepatitis and Regression of Fibrosis. Gastroenterology.

[140] Robertson A.G.N., Wiggins T., Robertson F.P., Huppler L., Doleman B., Harrison E.M., Hollyman M., Welbourn R. (2021). Perioperative mortality in bariatric surgery: Meta-analysis. Br. J. Surg..

[141] Arterburn D.E., Telem D.A., Kushner R.F., Courcoulas A.P. (2020). Benefits and Risks of Bariatric Surgery in Adults A Review. JAMA J. Am. Med. Assoc..

[142] Ohta M., Seki Y., Wong S.K.H., Wang C., Huang C.K., Aly A., Baijal M., Al-Sabah S., Udomsawaengsup S., Heo Y.S. (2019). Bariatric/Metabolic Surgery in the Asia-Pacific Region: APMBSS 2018 Survey. Obes. Surg..

[143] Panunzi S., Maltese S., Verrastro O., Labbate L., De Gaetano A., Pompili M., Capristo E., Bornstein S.R., Mingrone G. (2021). Pioglitazone and bariatric surgery are the most effective treatments for non-alcoholic steatohepatitis: A hierarchical network meta-analysis. Diabetes Obes. Metab..

[144] Collazo-Clavell M.L., Shah M. (2020). Common and Rare Complications of Bariatric Surgery. Endocrinol. Metab. Clin. N. Am..

[145] Kumar N., Choudhary N.S. (2015). Treating morbid obesity in cirrhosis: A quest of holy grail. World J. Hepatol..

[146] Eilenberg M., Langer F.B., Beer A., Trauner M., Prager G., Staufer K. (2018). Significant Liver-Related Morbidity After Bariatric Surgery and Its Reversal-a Case Series. Obes. Surg..

[147] Wai J.W., Fu C., Wong V.W.S. (2020). Confounding factors of non-invasive tests for nonalcoholic fatty liver disease. J. Gastroenterol..

